# Effect of Electronic Screening With Personalized Feedback on Adolescent Health Risk Behaviors in a Primary Care Setting: A Randomized Clinical Trial

**DOI:** 10.1001/jamanetworkopen.2019.3581

**Published:** 2019-05-10

**Authors:** Laura P. Richardson, Chuan Zhou, Elon Gersh, Heather Spielvogle, James A. Taylor, Carolyn A. McCarty

**Affiliations:** 1 Center for Child Health, Behavior, and Development, Seattle Children’s Research Institute, Seattle, Washington; 2 Department of Pediatrics, University of Washington, Seattle; 3 Department of Research and Translation, Orygen, the National Centre of Excellence in Youth Mental Health, Melbourne, Australia; 4 Centre for Youth Mental Health, University of Melbourne, Melbourne, Australia

## Abstract

**Importance:**

Health risk behaviors are a leading cause of morbidity during adolescence. Screening and counseling for health risk behaviors are recommended but infrequently performed.

**Objective:**

To test the effect of an electronic screening and feedback tool on clinician counseling and adolescent-reported health risk behaviors.

**Design, Setting, and Participants:**

A randomized clinical trial compared electronic screening and feedback on an intention-to-treat basis with usual care among 300 youths 13 to 18 years of age at 5 pediatric clinics in the Pacific Northwest. Outcomes were assessed via electronic survey at 1 day and 3 months after the initial visit. Study data collection occurred from March 13, 2015, to November 29, 2016, and statistical analysis was conducted between February 6, 2017, and June 20, 2018.

**Interventions:**

Youths in the intervention group (n = 147) received electronic screening and personalized feedback with clinician clinical decision support. Youths in the control group (n = 153) received standard screening and counseling as provided by their clinic.

**Main Outcomes and Measures:**

Youths’ report of receipt of counseling during the visit and risk behaviors at 3 months.

**Results:**

In the final study sample of 300 youths (intervention group, 75 girls and 72 boys; mean [SD] age, 14.5 [1.4 years]; and control group, 80 girls and 73 boys; mean [SD] age, 14.5 [1.4] years), 234 (78.0%) were aged 13 to 15 years. After adjusting for age, sex, and random effect of clinic, youths in the intervention group were more likely to receive counseling for each of their reported risk behaviors than were youths in the control group (adjusted rate ratio, 1.32; 95% CI, 1.07-1.63). Youths in the intervention group had a significantly greater reduction (β = –0.48; 95% CI, –0.89 to –0.02; *P* = .02) in their risk behavior scores at 3 months when compared with youths in the control group.

**Conclusions and Relevance:**

Electronic screening of health risk behavior with clinical decision support and motivational feedback to teens can improve care delivery and outcomes.

**Trial Registration:**

ClinicalTrials.gov identifier: NCT02360410

## Key Points

**Question:**

Does electronic risk screening with personalized feedback and clinical decision support increase clinician counseling and reduce risk behaviors in adolescents?

**Findings:**

In this randomized clinical trial of 300 adolescents randomly assigned to receive electronic screening with personalized feedback vs usual care in the context of a well-child care visit, youths who received the electronic screening intervention were more likely than controls to receive risk counseling. Three months after the visit, youths who received the electronic screening also had a significantly greater reduction than controls in their risk behavior scores.

**Meaning:**

Electronic screening tools that provide risk information to clinicians and motivational feedback to teens can improve care delivery and youth outcomes.

## Teaser

This randomized clinical trial tests the effect of an electronic screening and personalized feedback tool on clinician counseling and adolescent-reported health risk behaviors.

## Introduction

Health-compromising behaviors, such as risky sexual behaviors and substance use, often increase in adolescence and are associated with significant short-term and long-term morbidity.^1,2^ In recognition of the effect of these behaviors, screening is recommended by Bright Futures^3^ and the Guidelines for Adolescent Preventive Services,^4^ 2 leading US adolescent well-child care guidelines. However, despite the availability of guidelines and training materials, implementation of preventive screening remains low.^5,6,7^ Less than half of youths who are seen for well care are screened and even fewer receive counseling regarding risk behaviors.^8,9,10,11,12^

Several studies have been conducted to improve the delivery of counseling during well-adolescent care visits. The addition of standardized screening methods, including electronic screening tools, has been shown to increase detection and clinician discussion.^13,14^ A recent review article identified only 9 studies of screening for multiple risk behaviors and health care–based interventions with youth outcome data.^15^ There was considerable variation across the studies in the intensity of the interventions, the behaviors studied, and the outcomes. None of the studies incorporated electronic feedback for youths.

We conducted a study to examine the efficacy of a youth-facing electronic screening and personalized motivational feedback tool, the Check Yourself tool, which was designed to be administered prior to a well-child care visit to prime youths for discussions with their clinicians. The tool also includes clinical decision support for clinicians including a streamlined clinician summary report to encourage counseling for high-risk and moderate-risk behaviors. This study examines the efficacy of this tool in increasing youth-reported clinician counseling during the well-child care visit and improving health behavior outcomes 3 months after the visit.

## Methods

This was a randomized clinical parallel-group intervention study conducted in the Pacific Northwest of the United States (trial protocol in Supplement 1). Adolescent participants (13-18 years of age) and 1 parent per teen were recruited from 5 pediatric clinics in the Puget Sound Pediatric Research Network in western Washington state between March 13, 2015, and August 8, 2016, with follow-up through November 29, 2016. Clinics were invited to participate based on the size of the practice and the number of adolescent patients and were added to the study on a rolling basis with a goal of recruiting 60 youths per site for a total recruitment goal of 300 youths. The target sample size was determined by the study statistician to give 80% power to detect a difference in the primary risk behavior measure with 95% certainty. All procedures for recruitment were approved by the Western Institutional Review Board. Parental consent and youth assent were required for participants 13 to 17 years of age, while consent was obtained directly from 18-year-old participants. Assent and consent were obtained in writing from participants who completed study procedures in person and verbally for individuals who completed study procedures via telephone. Study results are presented following the Consolidated Standards of Reporting Trials (CONSORT) reporting guideline.

Adolescents with an upcoming well visit were invited by study staff to participate first via letter with an opt-out telephone number followed by a telephone call to assess eligibility and obtain consent and assent. Exclusion criteria included being outside the study age range, lacking telephone or internet access to complete surveys, having a sibling who was previously invited to participate, having unenrolled from the clinic or cancelled the well visit, or not speaking English. Participants were told that they would receive 1 of 2 versions of an electronic tool: version 1 would have feedback and a clinician report and version 2 would have no feedback and no report to the clinician.

For random allocation of participants, a statistician-developed computer-generated list of random numbers was entered into DatStat^16^ with 1:1 randomization stratified by age (13-15 or 16-18 years), sex, and clinic. Randomization was completed after participants completed consent and assent procedures with study staff. The participants were not explicitly told their assignment but they could potentially intuit it based on whether they received electronic feedback. Baseline data were collected by trained study staff either in person in a private setting or online with telephone support in advance of the youths arriving in the clinic. For all youths, study staff emphasized the importance of completing screening in a confidential setting.

### Intervention Procedures

Participants who received the intervention completed an electronic screening tool with personalized feedback and their clinician received clinical decision support via a clinician summary. The tool screened participants for protective factors and risk behaviors following a HEADSS (home, education, activities, depression, sexual activity, safety, and substance use) framework.^17^ In addition, the tool screened for specific nutritional behaviors, physical activity, and sleep. The personalized feedback, delivered as part of the same screening session based on integrated algorithms, was designed to motivate healthier behaviors and to encourage discussions with the clinician during the well visit. Specific types of feedback included comparison of reported behaviors with rates reported by peers or national guidelines, education about health risks, and tips for behavior change.^18^ Based on internal tracking data, the tool took an average of 15 minutes to complete.

The clinician summary report consisted of a dashboard that flagged youth behavior as low risk, moderate risk, or high risk within the following 6 categories: nutrition, activity, substance use, emotions, sexual activity, and safety. Below the dashboard, individual responses were provided for questions in each category. High-risk and moderate-risk behaviors were defined a priori based on health guidelines or expert consensus (eTable in Supplement 2). Study staff coordinated with clinic administrative staff to ensure that clinicians received a printed summary report prior to the visit.

### Control Procedures

Youths in the control group completed only baseline electronic screening. Clinicians of youths in the control group did not receive results of electronic screening. Clinics were encouraged to continue their standard preexisting health risk screening procedures for all youths regardless of study group throughout the study and not to alter their standard procedures for study participants. Standard well visit screening procedures varied by site, with some clinics using paper screeners at intake and others relying solely on clinician interview to identify health risks.

As patients were randomized on an individual basis, clinicians could care for both intervention and control patients. Prior to the onset of the study in each clinic, all clinicians in the clinics were invited to participate in a single 15-minute online training module orienting them to the tool, with a very brief overview of the tenets of motivational interviewing.

### Follow-up Surveys

Participants completed follow-up surveys 1 day and 3 months after their well visit either online or over the telephone with research assistant support. The 1-day follow-up survey asked about receipt of discussion and counseling to change behavior for each screened behavior and the youth’s level of motivation to change his or her behaviors. Questions for this section were adapted from the Adolescent Report of the Visit developed by Ozer and colleagues.^19^ At the 3-month follow-up survey, youths completed questionnaires assessing the same health risk behaviors as at baseline collected via an online survey tool, DatStat.^16^ All youths who reported suicidal ideation at the baseline screening or 3-month follow up assessment received a telephone call from a study clinician to assess risk and assist with connecting the youth with care, if needed.

### Statistical Analysis

Statistical analyses were conducted between February 6, 2017, and June 20, 2018. Data were uploaded into R, version 3.5.0^20^ for statistical analysis. Bivariate analyses were conducted to examine differences between youths in the control and intervention groups in demographics and baseline risks. Race and ethnicity were gathered directly from youths as part of intake screening to better describe participants to inform generalizability. There were 2 primary outcome measures for analyses determined a priori at the time of study design: receipt of counseling during the well visit and a summary score of health risk behaviors measured 3 months after the well visit.

Receipt of counseling, measured 1 day after the well visit, was defined as youth-reported receipt of clinician counseling to change a behavior toward better health. Poisson regression models compared the youths in the intervention and control groups regarding the total number of youth-reported moderate-risk and high-risk behaviors for which they reported receiving counseling. To ensure that the coefficient of group indicator captured the differences in rates of targeted counseling between groups, the overall number of moderate-risk and high-risk behaviors reported by youths was included as an offset variable in the Poisson models. To explore potential differential effects of the intervention on high-risk vs moderate-risk behaviors, secondary analyses were conducted examining the association between intervention status and rates of counseling for each of these categories of behavior. All regression analyses controlled for age at baseline and sex, consistent with the stratified randomization, and included a clinic-specific random effect to account for clustering within clinics.

The primary outcome measure for the assessment of risk behaviors at 3 months after the intervention was a summary score of all 13 assessed behaviors. The score was weighted based on tool-defined risk levels: high-risk behaviors were assigned a score of 2, moderate-risk behaviors a score of 1, and low-risk behaviors a score of 0 (eTable in Supplement 2). A total risk score was calculated for each youth by summing all individual risk scores, with a potential total range of 0 to 21. Linear regression methods were used to compare differences in youth-reported total risk score at 3 months in youths in the intervention group vs those in the control group controlling for the baseline risk score. We also conducted exploratory analyses to examine differential effects of the tool on high-risk vs moderate-risk behaviors using separate Poisson regression analyses to examine the total number of behaviors meeting each of these categories. Finally, to examine if there were stronger effects of the intervention on specific health risk behaviors, we conducted exploratory logistic regression analyses for each of the assessed behaviors. Because of concerns about estimate instability, logistic regression analyses were not conducted for behaviors reported by fewer than 10 youths per study group.

The control group was considered the reference group for all regression analyses. For mixed-effects Poisson and logistic regression models, we assessed statistical significance of estimates based on whether 95% CI for rate ratio or odds ratio included 1. For mixed-effects linear regression models, statistical significance was based on *P* < .05 calculated via the Satterthwaite *df* method.^21^ In secondary analyses examining individual behaviors, we used a conservative definition of *P* < .01 to reduce the likelihood of identifying a spurious relationship by chance.

## Results

**Figure 1.**
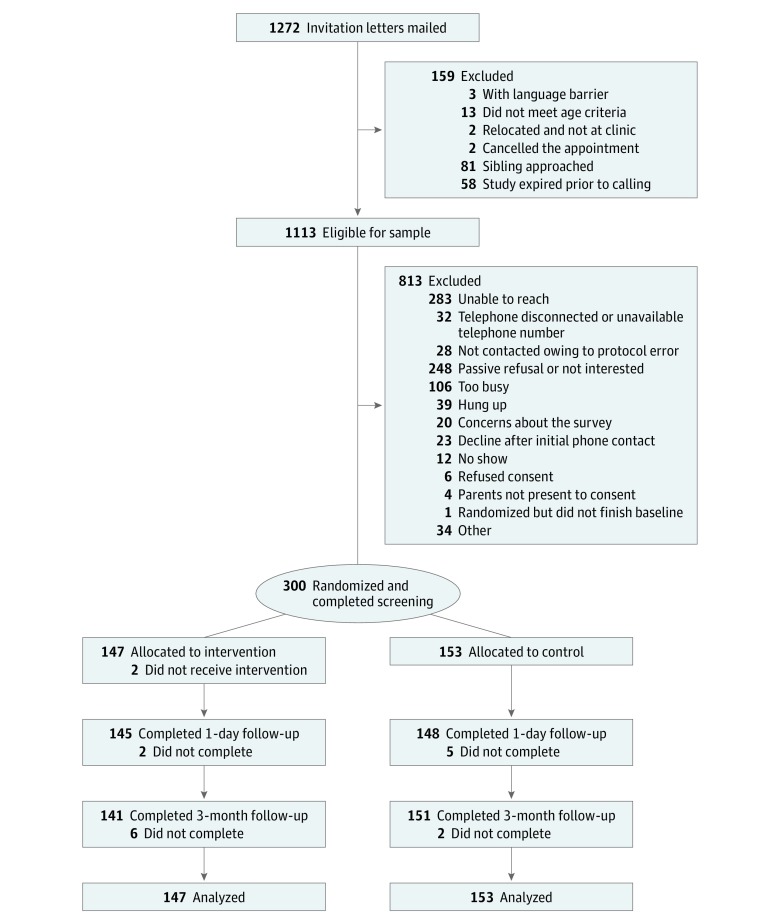
CONSORT Diagram

In total, 1272 adolescents were sent a letter inviting them to participate (Figure 1). The final study sample included 300 youths (intervention group, 75 girls and 72 boys; mean [SD] age, 14.5 [1.4 years]; and control group, 80 girls and 73 boys; mean [SD] age, 14.5 [1.4] years) who completed all consent and baseline procedures (27.0% of the eligible sample of 1113 youths); 147 individuals were randomized to the intervention group and 153 were randomized to the control group. Because of an error in implementation, 2 youths who were randomized to the intervention group did not receive feedback. They were retained in the intervention group for all analyses, consistent with an intention-to-treat protocol. The response rate was 97.7% (n = 293) for the 1-day follow-up and 97.3% (n = 292) for the 3-month follow-up.

### Baseline Demographics and Risk Assessment

There were no differences between the intervention and control groups in demographics, baseline risk score, or the number of baseline moderate-risk or high-risk behaviors (Table 1). A total of 155 participating youths (51.7%) were female. Youths predominantly identified as white (201 [67%), Asian (40 [13.3%]), and other or multiracial (46 [15.3%]). A total of 9 participants (3.0%) identified as Hispanic. A total of 234 youths (78.0%) were aged 13 to 15 years. A total of 125 youths (41.7%) lived in homes where at least 1 parent had a graduate or professional degree.

**Table 1.** Baseline Characteristics of the Sample^a^

Characteristic	Control Group (n = 153)	Intervention Group (n = 147)
Sex		
Male	73 (47.7)	72 (49.0)
Female	80 (52.3)	75 (51.0)
Age, mean (SD), y	14.5 (1.4)	14.5 (1.4)
Race/ethnicity		
White	99 (64.7)	102 (69.4)
Asian	24 (15.7)	16 (10.9)
Hispanic	3 (2.0)	6 (4.1)
African American	3 (2.0)	0
Native American	0	1 (0.7)
Other or multiracial	24 (15.7)	22 (15.0)
Parental educational level		
High school graduate or less	4 (2.6)	9/145 (6.2)
Technical school or some college	28 (18.3)	26/145 (17.9)
College graduate	56 (36.6)	50/145 (34.5)
Graduate or professional degree	65 (42.5)	60/145 (41.4)
Baseline risk score, mean (SD)	3.39 (2.27)	3.71 (2.79)
Reported risk behaviors at baseline, mean (SD), No.		
Moderate	2.17 (1.23)	2.18 (1.32)
High	0.61 (0.82)	0.76 (1.07)

^a^ Data are presented as number (percentage) of patients unless otherwise indicated.

A total of 285 youths (95.0%) had at least 1 health risk behavior at baseline. The distribution of risk behaviors was similar across study groups. Table 2 lists the reported risk behaviors in order of baseline frequency, with the most commonly reported risk behavior in the sample being low fruit and vegetable intake (control group, 125 of 153 [81.7%] vs intervention group, 114 of 147 [77.6%]) and the least frequent being unsafe sex (control group, 1 of 153 [0.7%] vs intervention group, 3 of 147 [2.0%]). The mean (SD) number of moderate-risk behaviors at baseline was 2.18 (1.32; median, 2; interquartile range, 1-3) for youths in the intervention group and 2.17 (1.23; median, 2; interquartile range, 1-3) for youths in the control group (*P* = .63). The mean (SD) number of high-risk behaviors at baseline was 0.76 (1.07; median, 0; interquartile range, 0-1) for youths in the intervention group and 0.61 (0.82; median, 0; interquartile range, 0-1) for those in the control group (*P* = .87) (Table 1).

**Table 2.** Moderate- and High-Risk Behavior Change From Baseline to 3 Months in Intervention and Control Groups

Health Risk Behavior	Youths, No. (%)				*P* Value^a^
	Control Group		Intervention Group		
	At Baseline (n = 153)	At 3 mo (n = 151)	At Baseline (n = 147)	At 3 mo (n = 141)	
Low fruit and vegetable intake	125 (81.7)	127 (84.1)	114 (77.6)	106 (75.2)	.41
High screen time	67 (43.8)	55 (36.4)	69 (46.9)	62 (44.0)	.37
Low sleep time	56 (36.6)	55 (36.4)	60 (40.8)	53 (37.6)	.68
Inconsistent helmet use	58 (37.9)	38 (25.2)	58 (39.5)	26 (18.4)	.01^b^
Low physical activity	40 (26.1)	44 (29.1)	42 (28.6)	31 (22.0)	.13
High sugary beverage intake	29 (19.0)	35 (23.2)	21 (14.3)	24 (17.0)	.93
Inconsistent seatbelt use	18 (11.8)	14 (9.3)	22 (15.0)	7 (5.0)	.006^b^
Marijuana or other drug use	14 (9.2)	11 (7.3)	16 (10.9)	10 (7.1)	.60
Alcohol use	6 (3.9)	9 (6.0)	12 (8.2)	9 (6.4)	NA^c^
Depression	5 (3.3)	11 (7.3)	9 (6.1)	12 (8.5)	NA^c^
Driving under influence	4 (2.6)	4 (2.6)	3 (2.0)	2 (1.4)	NA^c^
Unsafe sex	1 (0.7)	3 (2.0)	3 (2.0)	2 (1.4)	NA^c^
Tobacco use	2 (1.3)	3 (2.0)	4 (2.7)	2 (1.4)	NA^c^

Abbreviation: NA, not applicable.

^a^ Statistical significance was set at *P* < .01.

^b^ Based on likelihood ratio test comparing mixed-effects logistic regression models with or without period-by-group interaction. Both models controlled for random effects corresponding to within individual clustering.

^c^ Statistical comparison testing not conducted for variables with fewer than 10 participants at baseline in both the control and case samples.

### Clinician Counseling by Group

**Figure 2.**
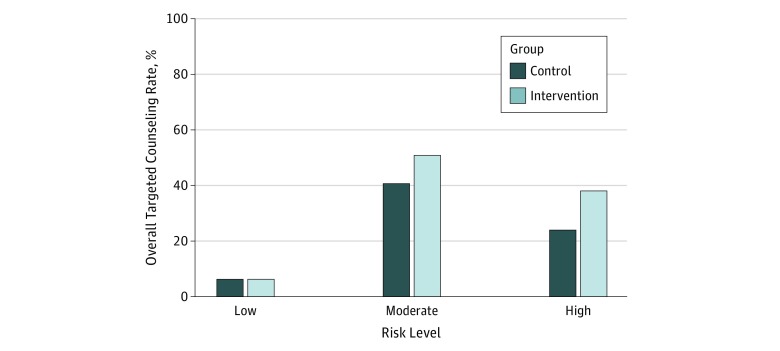
Rate of Risk Behaviors for Which Youths Received Counseling

Youths in the control reported 319 moderate-risk behaviors and received counseling for 130 of these behaviors (40.8%) during clinician visits; those in the intervention group reported 314 moderate-risk behaviors and received counseling for 160 of these behaviors (51.0%) during clinician visits (*P* < .001). Youths in the control group reported 87 high-risk behaviors and received counseling for 21 of these behaviors (24.1%) during the clinician visit; those in the intervention group reported 105 high-risk behaviors and received counseling for 40 of these behaviors (38.1%) during the clinician visit (*P* < .001). Youths in the intervention group were significantly more likely to receive counseling for their reported moderate-risk and high-risk behaviors than were those in the control group (adjusted rate ratio [aRR], 1.32; 95% CI, 1.07-1.63). To examine differential effect of the intervention on counseling for high-risk, moderate-risk, and low-risk behaviors, we also examined rates of counseling for youths in the intervention and control groups in each of these categories (Figure 2). Youths in the intervention group were 1.28 times more likely than those in the control group to report having received counseling for moderate-risk behaviors (aRR, 1.28; 95% CI, 1.02-1.62). For high-risk behaviors, the rate of counseling was 1.61 times higher among the intervention group than the control group, but this difference was not statistically significant (aRR, 1.61; 95% CI, 0.95-2.73). There were no significant differences in counseling for no-risk or low-risk behaviors between the intervention and control groups (aRR, 1.02; 95% CI, 0.77-1.36).

**Figure 3.**
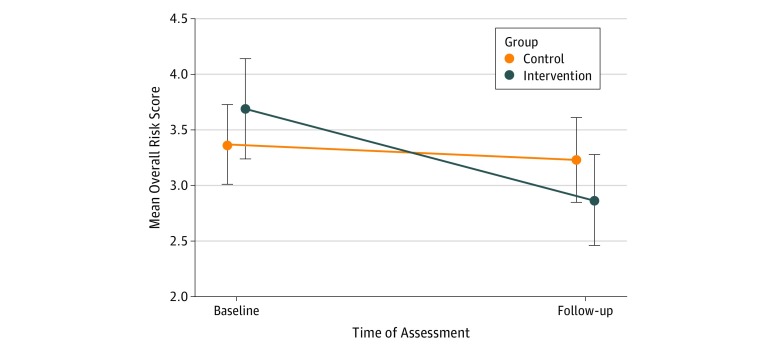
Change in Overall Risk Score from Baseline to 3 Months Comparing Intervention With Control Groups Vertical lines indicate 95% CIs.

The overall combined mean (SD) baseline risk score was 3.71 (2.79) in the intervention group and 3.39 (2.27) in the control group (*P* = .48) (Figure 3). At 3 months, the mean (SD) risk score for the intervention group was 2.89 (2.41), compared with 3.25 (2.37) for those in the control group (*P* = .08). On mixed-effects linear regression analysis, youths in the intervention group had a significantly greater decrease in risk behavior scores at 3 months compared with those in the control group (β = –0.48; 95% CI, –0.89 to –0.02; *P* = .02). When examining for effect modification by moderate-risk or high-risk behavior status, the intervention had a significant effect on reduction in the number of high-risk behaviors in the intervention group vs the control group (aRR, 0.61; 95% CI, 0.43-0.88), but not on the number of moderate-risk behaviors (aRR, 0.91; 95% CI, 0.78-1.07). In secondary analyses examining individual behaviors, significant reductions of behaviors in the intervention vs control groups were noted for only 2 behaviors: inconsistent helmet use (26 of 141 [18.4%] vs 38 of 151 [25.2%]) and inconsistent seatbelt use (7 of 141 [5.0%] vs 14 of 151 [9.3%]) (Table 2).

## Discussion

This study found that use of the electronic screening and personalized feedback tool increased the delivery of counseling and was associated with reduced overall rates of adolescent risk behaviors 3 months later. This finding suggests that electronic screening with integrated personalized feedback holds promise as a strategy for improving not only screening but also counseling for risk reduction and adolescent behavior change. To our knowledge, this is the only study that has examined the effect of a tool that simultaneously combines behavioral risk assessment with patient feedback and clinical decision support for clinicians on health risk behavior outcomes.

Adolescent health guidelines recommend screening across a broad range of behaviors in the context of preventive health visits. One key benefit of the tool is that it was designed to provide positive reinforcement, education, and tips for youths who were not reporting risk, thus ensuring that all youths receive some feedback on their behaviors. In addition, the clinician summary report was designed to provide clinical decision support by highlighting areas of reported risk to encourage clinicians to prioritize targeted discussion of higher-risk behaviors vs areas where behavior indicated low risk. The finding that youths in the intervention group were more likely than those in the control group to receive counseling for reported risk behaviors suggests that the tool was effective in helping clinicians prioritize counseling based on reported risk. Because clinicians do not discuss all behaviors with all patients, this approach may result in lower overall rates of counseling than interventions that target increased discussions of behaviors among all youths,^19^ but it may allow for more efficient use of time to address clinically relevant behaviors. Clinicians in our study completed a single 15-minute online training with basic motivational interviewing principles, compared with prior interventions that have used more intensive training^22,23,24^ and/or delivery of counseling by another clinician.^22,25^

### Limitations

This study has limitations. One key difference between our analytic approach and that of other studies is that one of our primary outcomes was a multirisk measure. The use of a combined measure allowed us to test across behaviors, which is important in a primary care sample in which the prevalence of any individual risk behavior might be low. Although there are many analytic benefits to the combined measure, one limitation of this approach is that it makes it more difficult to interpret in the context of specific health behaviors. We conducted secondary analyses of individual behaviors to allow for more ready interpretation of the intervention; however, for many behaviors the prevalence at baseline was too low to adequately assess the effect of the tool and thus we were limited in our ability to definitively draw conclusions on the effect of the tool on low-frequency behaviors. Helmet use, which is the only behavior that has shown improvements in 2 previous studies,^23,26^ was of adequate prevalence and showed significant improvement in our sample as well. Seatbelt use, which showed significant improvement in our sample, did not show consistent patterns of improvement in prior studies.^23,26^

Another limitation of this study was that few youths reported high-risk behaviors, which resulted in limited power to examine these outcomes. We believe that the low rate of behaviors is owing to the selection of youths seeking well care in pediatric primary care clinics. The rates of risk behavior prevalence in our study were similar those of to a more recent large-scale pediatric primary care screening study.^27^ Several factors may predispose pediatric primary care samples to lower rates of risk behavior. First, consistent with our study, in which 234 participants (78.0%) were in the age range of 13 to 15 years, prior research has shown that younger adolescents are up to 3 times more likely than older teens to have preventive visits.^28^ Because risk behaviors increase with age, their prevalence would be expected to be lower for a younger sample. Second, youths who are engaging in risk behaviors, such as sexual activity, may seek health care from nonpediatric settings in which there may be greater access to contraceptive care, such as school-based health clinics or family medicine settings. Using the same screening tool, another study found higher rates of high-risk behaviors in school-based health settings.^29^ Third, parents typically bring their children to well-child care visits, which may influence youths’ reporting of risk behaviors. Although we instructed all youths to complete the electronic screening tool in a confidential setting, it is possible that concerns about confidentiality led them to underreport some behaviors. Future studies should aim to better understand the prevalence of health risk behaviors in pediatric primary care settings, where screening is recommended, to inform the right level of interventions. In addition, regarding our tool, more work is needed in a larger sample of older adolescents and higher-risk youths to better understand its generalizability and effectiveness in these populations.

Other limitations include the fact that it is not possible to separate the effects of the feedback in the tool from those of clinician counseling because of the design of the study. As randomization occurred at the level of the patient, it is also possible that clinicians changed their practices with youths in the control group based on what they learned from working with youths in the intervention group. To the extent that this change occurred, it would introduce a conservative bias and make it less likely to detect a difference between groups. Also, this study was conducted among predominantly white low-risk youths seen for a well visit in primary care clinics in the Pacific Northwest and may not be generalizable to other, more diverse settings.

## Conclusions

Despite these limitations, this tool has the potential to significantly improve outcomes for youths. Health risk behaviors are the leading cause of morbidity and mortality in youths and across the life span, with even modest changes conferring potentially important long-term implications for the health of youths. Electronic screening with personalized feedback requires minimal training and clinician time for implementation and may be an effective strategy for delivering preventive and risk reduction counseling to youths.

## Notes

Supplement 1. **Trial Protocol**

Supplement 2. **eTable.** Risk Behaviors Included in Overall Summary Outcome Measure **eReferences**

Supplement 3. **Data Sharing Statement**
