# Supplementary material for: Effect of Electronic Screening With Personalized Feedback on Adolescent Health Risk Behaviors in a Primary Care Setting: A Randomized Clinical Trial
Source: JAMA Netw Open. 2019 May 10;2(5):e193581. doi: 10.1001/jamanetworkopen.2019.3581 (PMC6512281; doi:10.1001/jamanetworkopen.2019.3581)
Supplement: Supplement 1. — Trial Protocol [file jamanetwopen-2-e193581-s001.pdf]

Check Yourself Study Protocol

I. Purpose and Background

a. Specific scientific objective(s) of research

Aim 1: To examine the impact of a web-based, electronic Personalized Motivational Feedback tool (ePMF) on adolescent-perceived care during the well visit assessed 1-day post-visit. We will examine the impact of the web-based Check Yourself tool on youth-reported receipt of PCP delivered risk-reduction counseling. As an exploratory analysis, we will also explore the impact of Check Yourself on healthcare satisfaction. These effects will be tested against a usual care condition.

Aim 2: To examine the efficacy of the web-based Check Yourself tool in reducing adolescent health risk behaviors following the primary care visit. We will examine change in risk behavior score from baseline to 3 months post-visit in the Check Yourself group compared to usual care. Behaviors to be included in the score are alcohol use, drug use, risky sexual activity, depression, physical activity, sugar sweetened beverage consumption, fruit and vegetable consumption, and seatbelt and helmet use. As a secondary analysis, we will explore changes in rates of each of these individual variables for Check Yourself versus usual care from baseline to 3 months.

b. Background (purpose, lay language)

Adolescents have some of the highest rates of risk behaviors of all age groups and health behaviors developed in adolescence can persist into adulthood. Health risk behaviors, such as alcohol and other drug use, smoking, unsafe sexual activity, and poor nutrition and physical inactivity are among the most common causes of adolescent illness and premature death. These behaviors carry significant risks for subsequent disease, disability, and healthcare burden. Despite these risks, health risk screening in primary care is infrequently performed and results are rarely followed by targeted intervention.

Adolescents list primary care providers (PCPs) among the first people with whom they would consider discussing risk behaviors, and report greater satisfaction with care when PCPs discuss these sensitive topics with them. Thus, primary care visits present a key opportunity for improving the health of adolescents. However, to take advantage of this opportunity, health systems strategies are needed that can practically be implemented in the time-pressured environment of primary care.

Studies have shown that electronic health risk screening is feasible and efficient in clinical practice, increases adolescents' comfort with disclosure of behavior and encourages utilization of preventive health services. Based on this evidence, electronic multi-risk behavior screening tools are being developed for adolescent care settings. However, to reduce risk behaviors, screening needs to be linked to interventions. In response to the need for screening-linked interventions, our study team has developed a web-based, electronic Personalized Motivational Feedback tool (ePMF) which we refer to as the "Check Yourself tool" for the purposes of participant recruitment, enrollment, and participation. Based on motivational interviewing, a technique to mobilize personal change, Check Yourself is designed to promote healthy choices for the multiple behaviors relevant to adolescents.

The Check Yourself tool uses adolescent health screen responses, treatment guidelines, and national data on health risk behaviors to: 1) provide direct feedback to adolescents aimed at

increasing motivation to both prevent and reduce risk behaviors and 2) summarize youth-reported risk behaviors, goals, and consequences for Primary Care Providers (PCPs) in order to stimulate patient-provider discussions around risk reduction. After completing a brief (15 minute) health survey, adolescents receive electronic feedback which includes normative information, messages consistent with motivational interviewing (i.e., awareness and consequences, discrepancies), and goal setting strategies. PCPs can also receive specific information on patient responses that allow them to reinforce healthy behavior choices and implement further brief in-person interventions for moderate to high-risk adolescents. Thus, Check Yourself will provide a streamlined report with decision support for PCPs regarding recommended next steps in care based on evidence-based guidelines, and information regarding youth-reported risks, consequences, and goals in order to foster conversations that support motivation to change. In this study, we will evaluate the perceived impact and efficacy of Check Yourself among a sample a sample of adolescents aged 13-18 years drawn from the targeted primary care clinics using a groups who receives usual care as a comparison.

II. Criteria for Subject Selection

a. Sample Size

We will enroll approximately 300 adolescent (age 13-18)-parent dyads who have an appointment with a provider at one of five participating clinics,(see appendix 1) .

b. Gender of Subjects

This study does not have any gender-based restrictions. However, given that mothers are more frequently primary caregivers, we estimate the following gender distribution in our study population:

Adolescents: 150 Females and 150 Males
Parents: 240 Mothers and 60 Fathers

c. Age of Subjects (rationale if restrictions)

Participants will include adult *parents* (over the age of 18 years) and *adolescents* (age 13-18). As health risk behaviors typically begin in adolescence and persist into adulthood, we are targeting adolescents rather than children less than 13 years of age.

d. Racial and Ethnic Distribution

We will recruit an adolescent and caregiver sample that is approximately 75% non-Hispanic White, 5% African, 7% Asian, 9% Hispanic and 4% other racial groups. Participants will be drawn from five participating clinics (Appendix 1). The geographic area situating our study population consists of the following approximate ethnic and racial breakdown: 71% White, 5% African American, 11% Asian, 1% Native Hawaiian/Pacific Islander, 1% American Indian or Alaska Native, and 11% multiracial; 9% of individuals are of Hispanic ethic background.

e. Inclusion Criteria

Eligible parents will have a child 13 to 18 years old who has an appointment to see a provider at a participating clinic, and will be able to understand English or Spanish.

Eligible adolescents will be 13 to 18 years old, have an appointment to see a provider at a participating clinic, and will be able to understand English.

f. Exclusion Criteria

Parents will be excluded from the study if they do not speak English or Spanish; or if their child is ineligible for participation for the reasons given below.

Adolescents will be excluded from the study if they do not meet age requirements, have dis-enrolled or cancelled their appointment with the participating clinic, lack the means to complete follow-up interviews (i.e., has neither telephone nor internet access), have a sibling who has/is being enrolled in the study, and/or are not able to understand English. Our experience in the Seattle region is that, because of English Language Learning supports, inability to read or understand English is a barrier for <0.5% of adolescents. Thus, we do not anticipate that language restrictions will significantly limit the demographic distribution of our participants.

g. Vulnerable Subjects

This study's focus is on adolescent health care, and adolescents are study participants. We plan to enroll 300 adolescent subjects age 13-18. Most will be minors. Study activities for minor adolescents will be no different from study activities for 18-year-old adolescents. The risk/benefit ratio is the same for all adolescent participants regardless of age. Research staff will obtain oral consent from 18-year-old participants directly, without contacting a parent. For younger adolescents, research staff will first obtain oral parental consent before obtaining oral assent.

III. Methods and Procedures

a. Study Design

This study will evaluate the effect of Check Yourself on adolescent-perceived quality of care and health risk behaviors using a parallel, two-arm randomized-controlled study design. The study will take place at five clinics (see Addendum).

We anticipate that approximately 60% of youth approached will participate in the study and will therefore invite approximately 500 adolescent/parent dyads to participate in the Check Yourself app to yield 300 adolescent/parent dyad participants in our total sample. Upon enrollment, parent participants will be asked to complete a brief, 10-minute questionnaire web-based about their education/occupation, their adolescent's health history, and potential concerns about their adolescent's health risk behaviors. Adolescents who enroll in the study will be randomized to receive either the Check Yourself intervention or usual care (UC). Randomization will be carried out by the Study Data Manager under the guidance of the statistician, with randomization stratified by age (13-15 or 16-18), gender within each clinic to assure an even distribution of these variables. The randomization sequences will be placed into 4 stratum specific blocks in Datstat, a data collection platform for healthcare research which will be used by research assistants to determine whether adolescents receive Check Yourself or Usual Care following the consent process.

Following randomization, at baseline (T1), adolescents will be asked to complete a screening survey online or via a tablet including questions about guideline-recommended health risk behavior screening areas including substance use, nutrition, physical activity, sexual behavior, depressive symptoms, and safety. The content of the screener questions are current standard of care as recommended screening practices.

Adolescents who are assigned to the Check Yourself intervention will receive personalized motivational feedback following completion of the screening as part of the same online or

tablet session. Their PCPs will receive a provider report including specific information on the adolescent's responses that will allow them to reinforce healthy behavior choices and implement further brief in-person interventions for moderate to high risk adolescents. Providing feedback on screening results to the participant and provider is consistent with current standard of care. However, the electronic method of delivery is unique to this project.

Adolescents randomized to usual care will complete the screening survey for research purposes, but neither they nor their PCP will receive feedback about their health behaviors. PCPs will be advised to screen and counsel all patients as they would normally do under usual care conditions, consistent with the current standard of care.

In order to administer follow-up surveys, we will ask adolescents to provide an email address, cell phone number, or phone number. One day (T2) following their PCP appointment, all adolescent participants will be asked via email, text message or phone (based on their preference) to complete a survey about their health self-efficacy, healthcare climate, change readiness, and experiences and satisfaction with treatment during the appointment with their PCP. Three months (T3) following their PCP appointment, adolescent participants will be asked via email, text message or phone to complete assessment of the same risk behaviors evaluated at T1 including questions about peer norms of alcohol and marijuana use. These surveys are not standard of care because they are not collected as part of an appointment, however, the probability and magnitude of discomfort associated with the completion of these surveys is no greater than ordinarily encountered in daily life or during the performance of routine psychological examinations/tests or medical care. While they are collected solely for research outcome assessment, the assessment includes similar content to the initial screening.

b. Data Analysis and Data Monitoring

Data Analysis

Data from the web-based surveys will be uploaded in to Stata analytic software. Prior to analysis, all variables will be checked for validity, missingness, and distributional assumptions. Preliminary analyses required for variable construction, any required imputation of missing data, assessment of psychometric properties of scale scores, and assessment of the validity of study variables will be completed before analyses evaluating intervention effects.

We will employ intent-to-treat techniques for all analyses associated with the Check Yourself intervention. Prior to conducting regression analyses, we will evaluate the effectiveness of randomization by comparing the differences in demographics, parent measures, and health risk behaviors at baseline between Check Yourself and usual care using t-tests (continuous variables) or chi-squared tests (categorical variables). The relatively large sample size (150 per condition) and the use of stratified randomization will increase the likelihood that groups will be balanced at baseline. However, in the case that any of the above variables are unbalanced between randomization arms, we will include the unbalanced variable as a covariate in all regression analyses.

For our primary analysis, the outcome will be a summary health risk variable constructed from weighted key risk behaviors (as shown in the table below). The score will be weighted based on tool-defined risk levels: high-risk behaviors will be assigned a score of 2, and moderate-risk a score of 1. Participants who do not meet the criteria for moderate or high risk will be considered low risk for that behavior and given a risk score of 0. A total risk score will be calculated for each youth by summing all individual risk scores. Linear mixed regression models will be used to compare differences in youth-reported total risk score at 3

months in intervention versus control youth controlling for baseline total risk score, age at baseline, gender and the clustering by clinic.

As part of the primary outcome, we will also examine receipt of counseling, measured 1-day post well visit. Receipt of counseling will be defined as youth-reported receipt of provider counseling to change a behavior towards health. The total number of endorsed moderate and high risk behaviors for which the youth reported receiving counseling will be treated as count outcome, and compared using Poisson regression models between intervention and control youth. To ensure that the coefficient of group indicator captures the differences in rates of *targeted* counseling between arms, the overall number of moderate and high risk behaviors endorsed by youth will be included as an offset variable in the Poisson models.

To examine the differential effects of the intervention on reducing high versus moderate risk behaviors, secondary analyses will be conducted examining the association between intervention status and rates of counseling for each of these categories of behavior. Secondary analyses will also include linear and logistic mixed regression methods to assess differential changes in individual risk behaviors between intervention and control arms. All regression analyses will control for age at baseline and gender consistent with the stratified randomization. A clinic-specific random effect will be included in all regression models to account for clustering within clinics.

Table 1:

Primary Outcome Measures – Our primary outcome measures include a summary variable of health risk behaviors measured at 3 months post visit and the rate of receipt of counseling for endorsed behaviors during the well visit. The risk summary measure includes behaviors in the Check Yourself tool (last column of Table) weighted based on tool-defined risk levels (low-risk, medium-risk, and high-risk).

Secondary Outcome Measures – We will assess differential effects of the tool on high versus moderate risk behaviors using separate Poisson regression analyses to examine the total number of behaviors meeting each of these categories. To examine if there are stronger effects of the intervention on specific health risk behaviors, we will conduct exploratory logistic regression analyses for each of the assessed behaviors.

**Health Domain**	**Low Risk=0**	**Moderate Risk=1**	**High Risk=2**
**Eating/Nutrition**			
Sweetened beverage consumption (drinks/day)	0-1	2+	
Fruit and vegetable consumption (servings/day)	4+	0-3	
**Activity**			
Days physical activity (>60 min)	4+	0-3	

Screen time (hrs./day)	0-2	3+	
Sleep (hrs./night)	8+	0-7	

Tobacco Use

Tobacco Use	None	Any	

Safety

Seatbelt use	Always		Not Always
Bike helmet use	Always		Not Always
Drives drunk or high	No		Yes

Alcohol*

Alcohol Risk	**Low risk for frequency** (**Age 13-15:** 0 days/mo; **Age 16-17:** 0-1 days/mo, **Age 18:** 0-2 days/mo)<br><br>AND<br><br>**Low risk for quantity** (by gender)<br><br>(**Boys: Age 13:** 0-2 drinks; **Age 14-15:** 0-3 drinks; **Age 16-18:** 0-4 drinks)<br><br>(**Girls: Age 13-17:** 0-2 drinks; **Age 18:** 0-3 drinks)	**Moderate risk for frequency** (**Age 13-15:** no moderate use**; Age 16-17:** 2 days/mo; **Age 18:** 3 days/mo)<br><br>AND<br><br>**Low risk for quantity** (see Low risk column)	**High risk for frequency (Age 13-15:** 1+ days/mo; **Age 16-17:** 3+ days/mo; **Age 18:** 4+ days/mo)<br><br>OR<br><br>**High risk for quantity** (by gender)<br><br>**(Boys: Age 13:** 3+ drinks; **Age 14-15:** 4+ drinks; **Age 16-18:** 5+ drinks)<br><br>(**Girls: Age 13-17:** 3+ drinks; **Age 18:** 4+ drinks)

Marijuana or other drug use*

Marijuana frequency<br><br>(days /month)	0 days	1+ days	
Other drug use	None		Any

Sex*

Risky sexual behavior	Any birth control at last sex<br><br>AND<br><br>Always uses condoms/barrier methods		No birth control at last sex<br><br>OR<br><br>Does not always use condoms/barrier methods

Emotions

PHQ-9 score	0-9		10+

*In cases where multiple indicators were used to define risk behavior (alcohol, marijuana, sex), a subject is considered at high risk if ANY indicator was endorsed at high risk, a subject is considered at low risk if ALL indicators were endorsed as low risk. Subjects are considered moderate risk if they have at least one moderate risk indicator and no high risk indicators.

Data Monitoring

The proposed research involves no greater than minimal risk to participants.

Adverse Events. Project staff will be trained to identify potential adverse events and instructed to report them immediately to the principal investigators. Should any study participant or caregiver express concerns about the study or their participation or appear distressed during any study activities, the witnessing research team member will bring the matter to the attention of the principal investigators who will distinguish serious adverse events from non-serious adverse events. Serious adverse events will be documented and discussed with the IRB as soon as possible, and reported to the IRB within 48 hours. An annual report will be submitted to HRSA and the IRB summarizing all adverse events.

Data Quality Assurance and Confidentiality. Data quality assurance will be monitored on an ongoing basis by a designated study team member. She will each conduct routine protocol compliance checks to ensure that safety procedures, such as ensuring participant confidentiality and maintaining approved standards for data transport, are strictly adhered to at each site. All study data will be stored in password-protected computer files and identified with study IDs. Analytic data files will contain no identifying information. Other confidentiality issues are discussed in the prior section "Protection Against Risks."

HIPAA Compliance. All participants will be recruited from clinical sites, and as such their participation is subject to the Health Insurance Portability and Accountability Act of 1996 (HIPAA) privacy and security standards. All relevant staff has completed required HIPAA training and all research activities will be conducted in compliance with HIPAA standards. All study data will be securely stored and labeled as discussed on the "Data storage and confidentiality" section of this protocol, below. PCPs of participants assigned to the Check

Yourself intervention will receive feedback including specific information on the adolescent's responses that will allow them to reinforce healthy behavior choices and implement further brief in-person interventions for moderate to high-risk adolescents. Although a participant's responses to the health screener constitute protected health information generated from the research study, since the results are shared with the participant's PCP for treatment and/or health-monitoring purposes, this disclosure falls within an exception of the minimally necessary requirement of the Privacy Rule (Section 164.502).

c. Data storage and confidentiality

One month prior the adolescent's scheduled appointment with their PCP, participating clinics will generate reports with potential participants' contact information from via electronic scheduling tools which will be provided to study staff. We will collect the minimum amount of information necessary to identify potentially eligible participants from the participating clinics' electronic scheduling tools. We have obtained a full waiver of authorization under HIPAA to request a waiver of documentation of consent/assent and waiver of the requirement of a signature on the waiver for use and disclosure of Protected Health Information. We have previously received a partial HIPAA waiver to obtain contact information for individuals who have scheduled an appointment at the participating clinics. Data used include: parent/guardian name, adolescent name, address, and telephone number. Parents/guardians will be contacted and will be invited to participate in the study and provide consent for their child to participate and their child's name and contact information. If the adolescent is over 18, we will contact them directly and invite them to participate in the study and provide their consent and their parent/guardian's name, address, and telephone number. Contact and identifying information will be for study recruitment purposes will only be accessible to study staff with a direct need to access this information (e.g., research staff mailing letters and conducting phone screening). We will assign a unique study number to individuals for recruitment purposes. This study number is then used in study files, rather than subjects' names. We will then maintain and protect a linking file, which links study number to participants' names and other identifying information including participants' e-mail addresses and cell phone numbers which will be collected. This linking file will be stored in Seattle Children's Research Institute (SCRI) on a secure database on the SCRI network. Access to the SCRI network is controlled by valid, networked user accounts which include study researchers and staff. Identifying information used to recruit participants will be destroyed within 6 years of the end of the study.

SCRI is responsible for storage of data collected from participants. Study data for enrolled participants will be identified by study IDs and will also be retained in a SCRI secure database on the SCRI network. Analytic data files will contain no identifying information. Paper-based data will be stored in locked file cabinets, with all identifying information removed.

Subject demographic data will be recorded in a password-protected database. Completed questionnaire data will be collected through DatStat Illume, a platform used to develop secure, web-based surveys, and then exported from DatStat Illume into SPSS, Stata/SE, and SAS for analysis purposes. Data are stored on a secure SQL server, and are available to study investigators for queries, reports, and download for analysis.

Web-based surveys will be accessed by logging on to a secure server with security and data integrity violations obviated by requiring participants to log in with a password unrelated to any identifying information on any on-line page or database. All information transferred

between client and server machines will be secured using 128-bit encrypted Secure Sockets Layer. All data stored in the online repository will be encrypted using the official Advanced Encryption Standard algorithm. Protocols have been informed by prior internet-based studies conducted by study investigators.

For participants randomized to the intervention, their Check Yourself baseline feedback report will be printed by study or participating clinic staff and given to the participant's PCP prior to their appointment. Participant Check Yourself reports will be shared with participating clinic staff via a protected, designated fax machine available only to clinic staff and providers or through a secure server with security and data integrity violations obviated by requiring them to log in with a password unrelated to any identifying information related to the research participant. All information transferred between client and server machines will be secured using 128-bit encrypted Secure Sockets Layer. All data stored in the online repository will be encrypted using the official Advanced Encryption Standard algorithm. Protocols have been informed by prior internet-based studies conducted by study investigators.

For purposes of analysis and manuscript preparation, any paper data files will be maintained for up to 3 years after the project ends, after which all paper data will be disposed of via shredding. Secure, electronic data files will be kept for up to 6 years after the project ends on SCRI's secure network.

c. Measures by Construct and Method

Below are the measures used in the study, organized by construct and method.

**Measures and Constructs**	**Method**
**Demographics**	
Gender, age, race, household makeup, school, truancy, support, goals	T1
**Parent Concerns**	
Guidelines for Adolescent Preventative Services Parent Questionnaire: adolescent health risk behavior concerns.	PQ
**Quality of Care**	
Adolescent Report of the Visit: adolescent-report of the PCPs' delivery of risk behavior screening and counseling based on risk level.	T2
Client Satisfaction Questionnaire: adolescent self-report of general satisfaction with care, service quality, and the extent to which the visit met needs and intention to return.	T2
Health Care Climate Questionnaire: adolescent self-report of how adolescents feel about visits to their primary care giver.	T2
Interval Receipt of Care: adolescent self-report of extent of follow up care.	T3
**Behavior Change Variables**	
Health Self-Efficacy Scale: self-report of perceptions of confidence to initiate behaviors that achieve goals.	T2
Readiness to Change Rulers: Self-report used to assess adolescent motivation for change among specific behaviors.	T2, T3
Peer Use Norms Alcohol and Marijuana: Adolescent's estimate peer alcohol and marijuana use	T3

Health Behavior Variables	
Alcohol Consumption, Tobacco Use, and Marijuana Use: self-report based on items about typical quantity and frequency of drinking, smoking/using tobacco, and marijuana use (each calculated separately) over the past 30 days.	T1, T3
Fruit and Vegetable Screener: self-report of fruit and vegetable consumption.	T1, T3
Physical Activity Screener: self-report of physical activity.	T1, T3
Sexual Risk: self-report of sexual activity, birth control, and condom use.	T1, T3
Patient Health Questionnaire-9 (PHQ-9): self-report of DSM-V symptoms for depression.	T1, T3
Safety: self-report to assess behaviors that contribute to unintentional injuries (e.g., seat belt use, helmet use).	T1, T3

Key: PQ=Parent Questionnaire
T1=Adolescent Questionnaire at Time point 1 (Baseline)
T2=Adolescent Questionnaire at Time point 2 (1-day)
T3=Adolescent Questionnaire at Time point 3 (3-month)

IV. Risk/Benefit Assessment

a. Risk Category

This is a minimal risk study, with the probability and magnitude of discomfort no greater than ordinarily encountered in daily life or during the performance of routine psychological examinations/tests or medical care. We have received a Full Waiver of Authorization under HIPAA for waiver of documentation of consent and waiver of the requirement for signature on the authorization for use and disclosure of health information.

b. Potential Risks and Protection Against Them

RISKS: We foresee three potential risks to study participation. The first risk to participants is that the information provided on the questionnaires may not remain confidential, based on the fact that study instruments contain personal information about health related behaviors (i.e., alcohol use, depression). The PCP may choose to put risk behavior information in the adolescent participant's electronic medical record. It is also possible that adolescents may leave their computer screen with information from the online surveys open in their web-browser and someone else might see it. Although we will emphasize the importance of completing the survey in a private setting, adolescent participants may still complete it in an open setting (i.e., computer in the family room) and other family members might observe their responses.

The second risk is that we may discover suicidality, prompting the study researchers and staff to potentially breach confidentiality by notifying parents/guardians and care providers. Adolescents with concerns for suicidal ideation will be asked if in the past 3 months they have had serious thoughts about ending their life and if they have ever made a suicide attempt. Further management is described in the Protection against Risk section below.

The third risk is that email addresses of participants sent from SCRI could become public. To minimize this risk, Seattle Children's Hospital, the institution through which this research takes place, uses Accellion, which is FIPS 140-2 compliant, to send secure emails.

Protections against Risks: Participants will be fully informed of the potential risks of participation, alternate treatment options, and their right to discontinue study participation at any time. Specific steps we will take to reduce known risks are specified below:

1. *Confidentiality and Protections*: As part of the baseline and follow-up surveys (T1, T2 and T3) procedures, adolescents and caregivers will be informed that the adolescent should complete the assessment privately and adolescents will be instructed to exit the web browser at the completion of the survey. For participants randomized to the intervention, Check Yourself feedback at baseline will be shared with treating PCPs (described in study information sheet), but data collected during all follow-up assessments will be held confidential and will not be shared. The study information sheet will clarify the nature of data collected and whether data will be shared with treating PCPs. The study information sheet will also outline situations in which results may need to be shared with caregivers (i.e. suicidal thoughts). All materials included in the chart by PCPs will be considered part of confidential patient data and will be bound by customary restrictions on access to patient records. For example, Washington State law requires consent of the youth to release information regarding their mental health, sexual health, and alcohol and drug use. In the clinical sites for this study, it is standard practice for clinicians to review adolescent records before they are released to the parents. It should be noted that screening of health risks including depression, sexual activity, and alcohol use is a routine and recommended aspect of adolescent clinical care. All study data will be securely stored in either password-protected computer files or in locked file cabinets and identified with study IDs (see section III.c. for specific data security measures).

2. *Management of suicidality*. Regardless of intervention status, participants with a positive response on the items assessing suicidality will be flagged for further evaluation during their appointment with the PCP. Research staff will track all flagged youth to ensure that an assessment is completed by one of the study clinicians. A study clinician will call to evaluate any youth with a positive response on items assessing suicidality using a protocol that we have employed in our prior depression studies and will assist in connecting the youth with care based on level of assessed risk. The assessment consists of a semi-structured interview (including questions about pervasiveness of thoughts, impulsivity, presence of a plan and current supports) with guidance for assessing risk. All youth judged to be at higher than minimal risk will be encouraged to seek care and assisted with identifying an appropriate resource (PCP, mental health specialty care, emergency services) based on severity. Youth who are found to be actively suicidal will be assisted with receiving resources within 24 hours. Youth who are at low risk will be assisted with connecting with the PCP within 1-2 weeks. For youth <18 years old who are found to be at higher than minimal risk, the investigator will also speak with a parent/guardian to share recommendations and offer assistance in accessing care. Consent procedures will make clear the situations in which a caregiver would be notified using standard clinical language regarding danger to self or others. Arrangements will also be made to follow up with parents or young adult (e.g., 18-year old) participants to ensure that they do not need further assistance in arranging care. This protocol has functioned smoothly in each of our prior studies and overall youth and parents have been grateful for the information provided and additional assistance in receiving care.

c. Potential Benefits

Individual Benefit (other than remuneration): All participants may receive some satisfaction or indirect benefit from contributing to this research. Intervention youth will receive personalized feedback aimed at increasing their self-efficacy and motivation to reduce health

risk behaviors. PCPs will also receive multi-risk behavior screening results for these youth, and will be encouraged to discuss these results with youth during the visit.

Societal Benefit: Potentially preventable health-compromising behaviors are among the leading causes of morbidity and mortality in the adolescent age group. Screening and preventive interventions are recommended by multiple professional organizations, but are often not performed. The current study takes an innovative strategy of personalized feedback for adolescents and their PCPs in order to increase the delivery of risk reduction counseling and improve adolescent health outcomes. This project will be an important contribution towards in testing a strategy that can be broadly disseminated to reduce health compromising behaviors in primary care settings. It will enhance scientific knowledge, increase technical capacity for prevention/intervention (through Check Yourself) and is designed to improve clinical practice. Society will benefit from the testing of a youth-friendly personalized feedback tool that is prepared for use in healthcare settings. In this regard, the minor risks incurred are outweighed by the anticipated benefits.

d. Alternatives to Participation

Participation is voluntary and discontinuation is an option at any time during the study.

V. Recruitment and Consent

a. Recruitment Method

Staff from Seattle Children's Research Institute (SCRI) will recruit and enroll participants through a rolling recruitment process, organized by clinic.

Potential participants will be identified two to six weeks before their scheduled appointment via a data pull from the clinic scheduling software. Research staff will mail an Introductory Packet to the youth's home containing:

- Introductory letter signed by the clinic with an opt-out phone number
- Introductory flyer
- Study Information sheet

These documents are attached to this application.

We have received a partial waiver of authorization under HIPAA to obtain contact information for potential participants. Our procedure will be:

1. Contact the parent by phone (please see attached Phone Script).
2. Screen for eligibility, explain the study, and answer questions. If the parent wishes to participate, study staff will arrange a meeting with the parent and adolescent via telephone to review the study information sheet and obtain oral consent/assent.

"Adult adolescent" (i.e. 18 year old) recruitment: "Adult adolescents" will be approached before their parent. Study staff will attempt to contact the "adult adolescent" by phone. Once the "adult adolescent" is contacted, study staff will explain the study, give detailed information, and answer questions. If the "adult adolescent" wishes to participate, staff will arrange a meeting with the adult adolescent via telephone to obtain oral consent. Then the parent will be approached.

b. Waiver of Documentation of Consent/Assent and Waiver of Authorization

We are requesting a waiver of documentation of consent/assent and waiver of Authorization. This research study poses no more than minimal risk to research participants. The questions

asked in the online baseline screening survey are based on standard screening questions, typically administered during routine health care visits and considered standard of care. What differs from standard of care is the electronic administration of the screening questions and the personalized electronic feedback. We are requesting this waiver because, whenever possible, we intend to deliver the online screening survey and follow-up visits via the internet, rather than in-person. Thus, it would not be practicable to obtain written parental consent and assent if study visits are completed online.

Parent oral consent and permission and oral assent.

1. Oral parent consent and permission will be obtained over the telephone, prior to the youth's appointment at a mutually agreed upon time. The study information sheet will include the required elements of authorization under HIPAA.

Minor adolescent oral assent. Study staff will reach the minor adolescent by phone to explain the study. Assent will be obtained in conjunction with the oral parental consent and permission..

"Adult adolescent" oral consent will be obtained via telephone . "Adult adolescents" may participate in the study even if their parent does not participate.

c. Subject Capability

All potential subjects will have the capacity to give consent.

d. Subject Comprehension

Interested *parents* will have the opportunity to discuss the study with SCRI staff prior to enrollment. Throughout this conversation, SCRI staff will assess parental understanding of the project and of participation expectations by asking parents to use their own words to describe the project and their role in it. Efforts to clarify and simplify the research and parents' role will be prioritized. Additionally, staff will provide reminders that parents retain the right to withdraw from the study at any time. SCRI staff will invite questions from the parents, reiterate their right to refuse participation, and ask whether they are comfortable with participation.

Interested *adolescents* will also have the opportunity to discuss the study with SCRI staff prior to enrollment. Throughout this second conversation, SCRI staff will assess teens' understanding of the project and of participation expectations by asking them to describe the project in their own words. Staff will remind youth that they can withdraw from the study at any time without adverse consequences. SCRI staff will encourage questions, reiterate the right to refuse participation, and ask whether they are interested in participating.

e. Consent Forms

Consent forms for parents and adolescents are included in this submission bundle.

f. Documentation of Consent

Parents may consent orally and adolescents may provide oral assent. Documentation of oral consent and assent will be noted by research staff and kept in study files.

g. Costs to Subject

There are no study-related costs for any participants.

h. Payment for participation

Parents/Caregivers who complete Parent Questionnaire will each be given a $10 incentive. *Adolescents* who complete Baseline, 1-day and 3-month Questionnaires will be given a $20 incentive after each questionnaire (with a possible total of $60 per child and $70 per family).

i. HIPAA

SCRI requires that research participants provide authorization to use their protected health information in connection with research. SCRI's HIPAA form was previously attached to this submission. We have identified the information that will be collected from participating clinics and we will be providing protected health information to PCPs of participants assigned to the Check Yourself intervention including specific information on the adolescent's responses that will allow them to reinforce healthy behavior choices and implement further brief in-person interventions for moderate to high risk adolescents. Although a participant's responses to the health screening survey constitute protected health information generated from the research study, since the results are shared with the participant's PCP for treatment and/or health monitoring purposes, this disclosure appears to fall within an exception of the minimally necessary requirement of the Privacy Rule (Section 164.502). Thus, we are asking for a waiver of documentation of consent and waiver of the requirement of signature on the Authorization. As detailed above in the data storage section, all information will be collected through fully secure sites and all participants are coded to prevent identification of individuals.
