# Supplementary material for: Effect of Electronic Screening With Personalized Feedback on Adolescent Health Risk Behaviors in a Primary Care Setting: A Randomized Clinical Trial
Source: JAMA Netw Open. 2019 May 10;2(5):e193581. doi: 10.1001/jamanetworkopen.2019.3581 (PMC6512281; doi:10.1001/jamanetworkopen.2019.3581)
Supplement: Supplement 2. — eTable. Risk Behaviors Included in Overall Summary Outcome Measure eReferences [file jamanetwopen-2-e193581-s002.pdf]

Supplementary Online Content

Richardson LP, Zhou C, Gersh E, Spielvogle H, Taylor JA, McCarty CA. Effect of electronic screening with personalized feedback on adolescent health risk behaviors in a primary care setting: a randomized clinical trial. *JAMA Netw Open.* 2019;2(5):e193581. doi:10.1001/jamanetworkopen.2019.3581

eTable. Risk Behaviors Included in Overall Summary Outcome Measure
eReferences

This supplementary material has been provided by the authors to give readers additional information about their work.

**eTable.** Risk Behaviors Included in Overall Summary Outcome Measure			
	**Low (0)**	**Moderate (1)**	**High (2)**
**Nutrition**			
Fruits/vegetables per day	3+	0-2	
Sugared drinks per day	0-1	2+	
**Activity**			
Screen time hrs/day	0-2	3+	
Sleep hrs/night	8+	0-7	
Days/week physically active for at least 60 min	4+	0-3	
**Safety**			
Seat belt use	Always		Any other answer
Bike helmet use	Always		Any other answer
Drives drunk or high	No		Yes
**Alcohol & Drugs**			
Alcohol#	Low risk for both alcohol frequency and quantity	Moderate risk on alcohol frequency and low risk on quantity	High risk on either alcohol frequency or quantity
Other Substance Use	None	Marijuana, cigarettes/tobacco	Other drugs
**Sexual Health**			
Risky sexual behavior	Used birth control at last vaginal sex<br>AND<br>Always uses condom or barrier method with sex		No birth control with last vaginal sex.<br>OR<br>Does not always use condom or barrier method with sex
Uses barrier method with sex	Always		Any other answer
**Depression**			
Patient Health Questionnaire 9-item (PHQ-9)[1] score	<10		10+

* High-risk behaviors were defined a priori as those with near term risk of morbidity or mortality
# Alcohol risk definitions were based on NIAAA guideline age-specific drinking frequency criteria[2] and Donovan age- and gender-specific quantity criteria[3]. (Specific criteria: High risk (13-15 yo) = drinking >0 days/month, >2 drinks/episode (females,13 yo males) or >3 drinks/episode (males 14-15). High risk (16-17 yo) = drinking >2 days/month,>2 drinks/episode (females), or >4 drinks/episode (males). High risk (18 yo)= drinking >3 days/month,>3 drinks/episode (females), or >4 drinks/episode per episode (males). Moderate risk was based on days/month of drinking only. Youth with meeting high-risk quantity criteria were always categorized as high risk. Since any drinking was considered high risk in 13-

© 2019 Richardson LP et al. *JAMA Network Open.*

15 year olds, there was no moderate use category. For the other age categories moderate risk was defined as drinking 2 days/month (16-17yo) or 3 days/month (18 yo) based on NIAAA criteria.

eReferences

1. Kroenke K, Spitzer RL, Williams JB. The PHQ-9: validity of a brief depression severity measure. *J Gen Intern Med.* 2001;16(9):606-613.
2. National Institute of Alcohol Abuse and Alcoholism. Alcohol Screening and Brief Intervention for Youth. A Practitioner's Guide. https://pubs.niaaa.nih.gov/publications/Practitioner/YouthGuide/YouthGuide.pdf. Accessed December 10, 2018.
3. Donovan JE. Estimated blood alcohol concentrations for child and adolescent drinking and their implications for screening instruments. *Pediatrics.* 2009;123(6):e975-981.

© 2019 Richardson LP et al. *JAMA Network Open.*
