# Supplementary material for: Effect of Electronic Screening With Personalized Feedback on Adolescent Health Risk Behaviors in a Primary Care Setting: A Randomized Clinical Trial
Source: JAMA Netw Open. 2019 May 10;2(5):e193581. doi: 10.1001/jamanetworkopen.2019.3581 (PMC6512281; doi:10.1001/jamanetworkopen.2019.3581)
Supplement: Supplement 3. — Data Sharing Statement [file jamanetwopen-2-e193581-s003.pdf]

Data Sharing Statement

Richardson. Effect of Electronic Screening With Personalized Feedback on Adolescent Health Risk Behaviors in a Primary Care Setting. *JAMA Netw Open*. Published May 10, 2019.
10.1001/jamanetworkopen.2019.3581

Data
Data available: No
